# The Queen's Jubilee

**Published:** 1886-12-11

**Authors:** 


### THE QUEEN'S JUBILEE:

### HOW WILL THE HOSPITALS CELEBRATE IT?

*The People Unanimous: The Spectacle Unique.*

NEXT year we hope to see what the world has hardly ever seen, the wonderful spectacle of an unanimous people. A great nation, several nations, many different "kindreds, peoples, and tongues," all united as the heart of one man. It is a strange and overpowering, sometimes even a terrible sight to see vast masses of people moved, absorbed, carried away by one common impulse. Such a spectacle, if the Queen's life be preserved, the world will witness next year. How profoundly thankful must every wholesome-hearted Briton be to think that there is at least one subject in which he can entirely agree with every other honest fellow countryman. Our endless controversies—religious, political, social and general – become sometimes wearisome, nauseous, disgusting. We sigh for peace: we long for quiet: we pray for agreement.

*The Wolf and the Lamb.*

Our peaceful aspirations will be satisfied for a brief season during the year which is about to open upon us. "The wolf will dwell with the lamb." The zealous Radical will greet the sober Conservative: the Churchman of "high" proclivities will clasp hands with his brother of "Evangelical" tendencies, and both will embrace the relenting Nonconformist. The prince will look kindly on the peasant, and the peasant hopefully to the prince The Queen, the mother of all her people, will regard them with the feelings of a mother, and will think of the poor and the suffering with peculiar tenderness; whilst the people themselves, remembering her fifty years' reign of unbroken justice, virtue and benevolence, will render to her, in the sight of heaven and earth, that loyal homage which is the offspring of love alone.

*The Hospitals: Together or Apart.*

The hospitals are preparing to play their part in the general movement of the people. Will there be unity among them, or will there be separation and isolation? If ever an event could be imagined when concerted action would be appropriate, it is surely the coming jubilee. The nature of the occasion demands it, the circumstances of the hospitals demand it, and the public feeling will demand, if it does not insist upon it. A movement in concert—in perfect accord and union: the hospitals have never yet made such a movement. They are like individuals, their own concerns loom too largely in their eyes. They cannot see that the interests of one are the interests of all; that each of them is but a part of one grand commonwealth, and that the well-being of the whole means the prosperity of each.

*The Folly of Isolation.*

What vast sums of money have been left for charitable uses by benevolent persons in past ages! Yet, in many cases the money has been a hindrance rather than a help. Some institutions, like some young men, have had enough money bequeathed to them to save them from starvation, and too much to render them self-reliant and efficient. There are instances of endowed hospitals which are almost dying of inanition; whilst, on the other hand, there are purely voluntary institutions, which are in the front ranks of efficiency and vigour. If the hospitals were in the habit of acting in concert, such things could not be: the wealth would then go to those who could use it, the tools to those who could handle them. We cannot suppose that pious founders, as a rule, had any intention save that of leaving their money in such a way as that the utmost possible value should be got out of it in the interests of the suffering poor. By concert those intentions would have been fulfilled; by isolation they are frustrated and defeated.

*The future shall be better than the past.*

But whatever may have been the case in the past, there should be a determination that in the present and future no such fatal errors shall be committed. There are still pious founders, benevolent men of wealth, and a generous public. It may surprise many, but it is nevertheless quite true, that the proportion of voluntary contributions to hospital needs is greater now than it ever was before, and has risen rapidly in the last ten years. This will be seen on reference to the article on the subject which appears elsewhere. During that period great efforts have been put forth to bring the hospitals into more intimate union, to help them to reform their administration, and to equalise and minimise their expenditure; to diminish the number of useless institutions already existing, and to prevent the establishment of new ones of the same character. These efforts, it will be seen, have been largely successful, and the result has been that in spite of a prolonged period of profound commercial depression, the public interest in hospitals has steadily increased.

*One and All: "A Long Pull and a Strong Pull"*

It is very natural that charitable institutions should look forward to an occasion like that of the Queen's Jubilee with "great expectations." Men's hearts will be full of sympathy for the sick and sorrowing, and struggling hospitals, which are conscious that their work is altogether and entirely good, may well be excused if they strive to take advantage of the rising tide of benevolence and to float on the crest of the wave into a secure haven of financial prosperity. So far from blaming, we approve of their laudable desire. But what we wish to see is not one institution, or two or three, entering the harbour on a right royal tide of prosperity, whilst scores of others are struggling out at sea or foundering on some distant rock. We want the whole fleet to be present at the grand celebration, and each one to have its due share of the good things of the gracious period. The Hospitals Week, which probably brought more than £10,000 additional income to the hospitals on Hospital Sunday, is proof positive of the value of combined effort by hospital managers. There can be no doubt that £1,000,000 sterling might be raised by a Jubilee Fund if all the London hospitals would combine, and such a sum is all these hospitals require to put them once and for all in a financially strong position. Sir Edmund Currie has given notice of his intention to bring this idea before his colleagues at the Mansion House, and who can doubt the result? As we said before, we think the occasion demands union and concert, and we fervently hope that some of the leading hospitals will take early steps to promote a movement which shall be alike worthy of the royal Jubilee and of the noble institutions whose interests are so deeply concerned. Such a movement should not be confined to the Metropolitan hospitals, but every large town should form a representative Jubilee Hospital Committee of all the charities, and so secure the whole of the local hospitals from ever coming on the rates. To encourage such an effort, Parliament might well be asked to exempt all voluntary hospitals from rating for the future, whilst providing for the registration of all new hospitals for all time coming.

